# Associations between Coffee Products and Breast Cancer Risk: a Case-Control study in Hong Kong Chinese Women

**DOI:** 10.1038/s41598-019-49205-x

**Published:** 2019-09-03

**Authors:** Priscilla Ming Yi Lee, Wing Cheong Chan, Carol Chi-hei Kwok, Cherry Wu, Sze-hong Law, Koon-ho Tsang, Wai-cho Yu, Yiu-cheong Yeung, Lydia Dai Jia Chang, Carmen Ka Man Wong, Feng Wang, Lap Ah Tse

**Affiliations:** 1 The Jockey Club School of Public Health and Primary Care, Faculty of Medicine, The Chinese University of Hong Kong, Hong Kong, People’s Republic of China; 2 Department of Surgery, North District Hospital, Hong Kong, People’s Republic of China; 3 Department of Oncology, Princess Margaret Hospital, Hong Kong, People’s Republic of China; 4 Department of Pathology, North District Hospital, Hong Kong, People’s Republic of China; 5 Department of Surgery, Yan Chai Hospital, Hong Kong, People’s Republic of China; 6 Department of Pathology, Yan Chai Hospital, Hong Kong, People’s Republic of China; 7 Department of Medicine, Princess Margaret Hospital, Hong Kong, People’s Republic of China; 8 Department of Biological Sciences, University of Toronto Scarborough, Toronto, Canada

**Keywords:** Epidemiology, Breast cancer

## Abstract

Coffee contains caffeine and diterpenes that were associated with decreased breast cancer risk, but results remained inconsistent. The study purpose was to investigate the associations between coffee products and breast cancer risk among Hong Kong Chinese women. We conducted a hospital-based case-control study in three public hospitals. 2169 Chinese women aged 24–84 years old were interviewed using a standardized questionnaire with questions asking types, cups and duration on coffee drinking. We used unconditional multivariate logistic regression to calculate the adjusted odds ratio (AOR) and 95% confidence interval (95% CI) for breast cancer risk with different coffee products. 238 (20.6%) cases and 179 (17.7%) controls are habitual coffee drinkers. No association was found between overall coffee drinking and breast cancer risk. Compared to the non-habitual coffee drinkers, women who consumed instant coffee (AOR = 1.50, 95% CI = 1.10–2.03) were significantly associated with an increased breast cancer risk. Women who drank brewed coffee (AOR = 0.48, 95% CI = 0.28–0.82) were negatively associated with breast cancer risk. A positive association between instant coffee and breast cancer risk was observed, contradicted to the outcomes of drinking brewed coffee. Larger studies are warranted to ascertain the role of different types of coffee products in breast cancer risk.

## Introduction

Globally, breast cancer is the most common cancer in women, and their dietary habits are among the most important factors contributing to the etiology of breast cancer^1^. In Hong Kong, coffee consumption is becoming more popular among local Chinese people. Statistics from Hong Kong Statista showed that the revenue of the coffee market increased by more than twice from 119 million USD in 2010 to 279 million USD in 2018, especially for that of the instant coffee^2^. Coffee consumption was classified as “possibly carcinogenic to humans” in 1991 but it has now been downgraded to “not classifiable as to carcinogenicity” in 1996 by the World Health Organization’s International Agency for Research on Cancer^3^. The association between coffee consumption and the risk of developing breast cancer has been investigated since the 1970s, but the evidence from epidemiological studies remains controversial^4–9^. Additionally, the composition of coffee is different among a variety of coffee products, such as instant coffee and brewed coffee, so these products may contribute differently to breast cancer risk.

A recent meta-analysis of 13 prospective studies did not show any significant association between coffee consumption and the risk of breast cancer, but an inverse relationship was found among postmenopausal women (relative risk = 0.90, 95% confidence interval (95% CI): 0.82–0.99)^10^. A prospective cohort study with 22 years of follow-up in Boston also demonstrated an inverse association of caffeine consumption, and the association was stronger with estrogen-receptor positive and progesterone-receptor positive breast cancer than that with the estrogen-receptor negative and progesterone-receptor negative breast cancer^4^. This inverse association may be caused by different coffee compounds and chemicals including caffeine, diterpene and polyphenols, which may influence breast cancer mechanisms. Coffee is the major dietary source of caffeine, and previous *in vitro* studies suggested that caffeine may bind the estrogen hormones through increased levels of sex hormone-binding globulin among post-menopausal women^11^. Thus, caffeine consumption may lower the levels of circulating sex hormones, thereby reduces the risk of breast cancer.

Different coffee products may also contain various levels of sugars and food additives, which may affect the rising trend of breast cancer in different ways. A majority of coffee consumption in Hong Kong is from instant coffee, while brewed coffee is the most popular choice in Western countries^2^. The toxicity of instant coffee in the Hong Kong market was tested by the Vitargent (International) Biotechnology Limited who followed the international standards (the CODEX Alimentarius of the WHO&FAO, European Union, and Food and Drug Administration of the United State and Japan)^12^. They found that more than 30% of instant coffee samples failed to achieve the standard in one or more of the following criteria regarding the acute toxicity, chronic toxicity and banned ingredients of instant coffee products (e.g., the chemicals disrupted the estrogenic endocrine system)^12^. However, there currently remains a lack of knowledge on the relationship between different coffee products and breast cancer risk in women. It is also important to note that the differences in coffee habits between Asian and Western countries. The highest level of coffee consumption in most of the studies conducted in Western countries was ≥4 cups per day, whereas the highest level in Asian countries was only ≥1 cup(s) per day^2^, thus the coffee consumption in Western countries and its contribution to breast cancer are not applicable to the Hong Kong population. To address this information, this study aims to investigate the associations between different coffee products (mainly focusing on instant coffee and brewed coffee) and breast cancer risk among Hong Kong Chinese women.

## Results

One thousand one hundred and fifty-six newly diagnosed breast cancer cases and 1,013 controls were recruited in this study with a response rate of 88.4% and 89.5%, respectively. As shown in Table 1, breast cancer cases were older than controls, more likely to be overweight and had a family history of breast cancer. The pattern of educational attainment, previous history of benign breast diseases, menopausal status, shift work experience, tobacco smoking, alcohol consumption and tea consumption, and deep fried food and green vegetable consumption was similar between the cases and controls.

**Table 1:** Distribution of selected characteristics of 1 156 cases and 1 013 controls among Hong Kong women.

Characteristic	Controls (*N* = 1 013)	Cases (*N* = 1 156)	*p-*value
Age, Mean ± SD	54.25 ± 11.7	56.53 ± 11.8	<0.01
Educational attainment, n (%)			
Primary or below	364 (35.9)	429 (37.1)	
Secondary	529 (52.2)	579 (50.1)	
Tertiary	77 (7.6)	78 (6.7)	
Unknown	43 (4.2)	70 (6.1)	0.59
Family breast cancer history, n (%)			
No	972 (96.0)	1031 (89.2)	
Yes	41 (4.0)	125 (10.8)	<0.01
Previous history of benign breast diseases, n (%)			
No	913 (90.1)	1017 (88.0)	
Yes	100 (9.9)	139 (12.0)	0.11
Menopausal status			
Pre-menopausal	341 (33.7)	388 (33.6)	
Post-menopausal	616 (60.8)	695 (60.1)	
Unknown	56 (5.5)	73 (6.3)	0.93
Body mass index, n (%)			
Normal (18.5–24.9)	541 (53.4)	630(54.5)	
Underweight (<18.5)	83 (8.7)	56 (4.8)	
Overweight (≥25.0)	228 (22.5)	399 (34.5)	
Unknown	161 (15.9)	71 (6.1)	<0.01
Shift work, n (%)			
No	861 (85.0)	1007 (87.1)	
Yes	152 (15.0)	149 (12.9)	0.16
Tobacco smoking, n (%)			
Never	933 (92.1)	1077 (93.2)	
Former	34 (3.4)	37 (3.2)	
Current	46 (4.5)	42 (3.6)	0.55
Alcohol consumption, n (%)			
Non users	969 (95.7)	1098 (95.0)	
Users	44 (4.3)	58 (5.0)	0.46
Tea consumption, n (%)			
Non users	434 (42.8)	467 (40.4)	
Users	579 (57.2)	689 (59.6)	0.25
Deep Fried food consumption, n (%)			
Less than once per month	453 (44.7)	518 (44.8)	
Once per month	381 (37.6)	429 (37.1)	
Once per week	177 (17.5)	208 (18.0)	0.94
Green vegetable consumption, n (%)			
Less than 3 times per week	110 (10.9)	128 (11.1)	
4–6 times per week	90 (8.9)	128 (11.1)	
Everyday	813 (80.3)	900 (77.9)	0.22

The distribution of specific coffee products and the associations with breast cancer is summarized in Table 2. A total of 239 (20.7%) cases and 179 (17.7%) controls were habitual coffee drinkers. Among them, 161 (13.9%) cases and 128 (12.6) controls consumed at least one cup of coffee per day. No association was observed between habitual coffee drinking or the number of cups of coffee consumption with breast cancer risk. However, subgroup analysis by different coffee products showed that breast cancer risk was negatively associated with habitual brewed coffee consumption (AOR = 0.48, 95% CI = 0.28–0.82) (Table 2), showing an exposure-response relationship with increasing cups of brewed coffee (p for trend < 0.01). Women who drank coffee served in restaurants at least once a week showed an even lower OR of the risk of breast cancer (AOR = 0.23, 95% CI = 0.08–0.65, p for trend < 0.01) (Table 3). In contrast, women who habitually drank instant coffee had an 41% increased risk of breast cancer (Table 2), showing a positive gradient of breast cancer risk with increasing consumption of instant coffee (p for trend = 0.02). The risk of breast cancer increased 1.51 fold among the habitual consumption of two-in-one or three-in-one instant coffee (Table 3). Furthermore, compared with women who habitually drank brewed coffee, women who drank instant coffee have an increased odds of breast cancer (AOR = 2.77, 95% CI = 1.49–5.13) after adjustment of same confounding factors as those are listed in Table 2 (Table 4).

**Table 2:** Distribution of specific coffee product among coffee consumption and its association with breast cancer risk^a^.

Variables	Controls (*N* = 1 013)	Cases (*N* = 1 156)	Crude OR (95% CI)^bc^	Adjusted OR (95% CI)^bcd^
Coffee drinking, n (%)	179 (17.7)	238 (20.6)	1.21 (0.97–1.50)	1.14 (0.88–1.47)
Instant coffee	113 (11.2)	182 (15.7)	**1.52** (**1.18–1.99)**	**1.50** (**1.10–2.03)**
Two/Three in one	15 (1.5)	37 (3.2)	**2.24** (**1.22–4.11)**	**2.52** (**1.24–5.13)**
Brewed coffee	59 (5.8)	43 (3.7)	**0.57** (**0.36–0.90)**	**0.48** (**0.28–0.82)**
Homemade coffee	25 (2.5)	26 (2.2)	0.86 (0.45–1.65)	0.67 (0.31–1.43)
Restaurant coffee	36 (3.6)	17 (1.5)	**0.39** (**0.20–0.76)**	**0.33** (**0.16–0.69)**
Others^e^	19 (1.9)	26 (2.2)	1.24 (0.68–2.26)	1.17 (0.60–2.28)

^a^Participants with missing values were excluded from the analysis.

^b^The reference group was defined as the participants who did not have a habitual coffee consumption (i.e., coffee drinking vs. non-coffee drinking; instant coffee drinking vs. non- coffee drinking). ^c^Participants who drank both instant coffee and brewed coffee were excluded in the logistic regression models. ^d^Adjusted for age at interview, educational attainment, family breast cancer history, previous history of benign breast diseases, body mass index (BMI), shift work experiences, smoking status, alcohol and tea drinking consumption, and deep fried food and green vegetable consumption. ^e^Other coffee referred to those who drank decaffeinated coffee or had no preferable coffee products.

**Table 3:** Distribution of number of cups of coffee products and its association with breast cancer risk^a^.

Cups of coffee drinking per day, n (%)	Controls (*N* = 1 013)	Cases (*N* = 1 156)	Crude OR (95% CI)^bc^	Adjusted OR (95% CI)^bcd^
**Overall coffee**				
Non-habitual coffee drinker	834 (82.3)	918 (79.4)	1.00	1.00
<1 cup/day	51 (5.0)	77 (6.7)	1.37 (0.95–1.98)	1.22 (0.80–1.85)
≥1 cup/day	128 (12.6)	161 (13.9)	1.14 (0.89–1.47)	1.09 (0.81–1.46)
**Instant coffee**				
Non-habitual coffee drinker	900 (88.8)	974 (84.3)	1.00	1.00
<1 cup/day	37 (3.7)	63 (5.4)	**1.55 (1.02–2.35)**	1.36 (0.85–2.17)
≥1 cup/day	76 (7.5)	119 (10.3)	**1.42 (1.05–1.93)**	**1.45 (1.02–2.06)**
Two/Three in one				
Non-habitual coffee drinker	998 (98.5)	1 119 (96.8)	1.00	1.00
<1 cup/day	4 (0.4)	10 (0.9)	2.27 (0.71–7.27)	2.91 (0.61–13.91)
≥1 cup/day	11(1.1)	27 (2.3)	**2.23 (1.10–4.52)**	**2.51 (1.14–5.57)**
**Brewed coffee**				
Non-habitual coffee drinker	954 (94.2)	1 112 (96.3)	1.00	1.00
<1 cup/day	24 (2.4)	25 (2.2)	0.95 (0.54–1.67)	0.67 (0.35–1.29)
≥1 cup/day	35 (3.5)	18 (1.6)	**0.47 (0.26–0.83)**	**0.40 (0.20–0.76)**
Homemade coffee				
Non-habitual coffee drinker	889 (97.7)	902 (97.5)	1.00	1.00
<1 cup/day	10 (1.0)	13 (1.1)	1.18 (0.52–2.71)	0.93 (0.37–2.33)
≥1 cup/day	15 (1.5)	13 (1.1)	0.79 (0.37–1.66)	0.65 (0.27–1.60)
Restaurant coffee				
Non-habitual coffee drinker	977 (96.4)	1 139 (98.5)	1.00	1.00
<1 cup/day	17 (1.7)	12 (1.0)	0.64 (0.30–1.35)	0.44 (0.19–1.03)
≥1 cup/day	19 (1.9)	5 (0.4)	**0.24 (0.09–0.64)**	**0.23 (0.08–0.65)**
**Others** ^**e**^				
Non-habitual coffee drinker	897 (98.2)	898 (97.4)	1.00	1.00
<1 cup/day	6 (0.6)	12 (1.0)	1.82 (0.68–4.86)	2.30 (0.69–7.64)
≥1 cup/day	13 (1.3)	14 (1.2)	0.98 (0.46–2.09)	0.80 (0.35–1.80)

^a^Participants with missing values were excluded from the analysis.

^b^The reference group was defined as the participants who did not have a habitual coffee consumption (i.e., coffee drinking vs. non-coffee drinking; instant coffee drinking vs. non- coffee drinking).

^c^Participants who drank both instant coffee and brewed coffee were excluded in the logistic regression models.

^d^Adjusted for age at interview, educational attainment, family breast cancer history, previous history of benign breast diseases, body mass index (BMI), shift work experiences, smoking status, alcohol and tea drinking consumption, and deep fried food and green vegetable consumption. ^e^Other coffee referred to those who drank decaffeinated coffee or had no preferable coffee products.

**Table 4:** Associations between controls and breast cancers in brewed coffee and instant coffee consumption.

Variables	Controls (*N* = 1 013)	Cases (*N* = 1 156)	Crude OR (95% CI)^bc^	Adjusted OR (95% CI)^cd^
Brewed coffee	59 (5.8)	26 (2.2)	1.00^a^	1.00^a^
Instant coffee	113 (11.2)	182 (15.7)	**2.55 (1.52–4.28)**	**2.77 (1.49–5.13)**

^a^The reference group was defined as the participants who have habitual brewed coffee consumption.

^b^Using brewed coffee as reference group and no other variables were adjusted in the model.

^c^Participants who drank both instant coffee and brewed coffee were excluded in the logistic regression models. ^d^Adjusted for age at interview, educational attainment, family breast cancer history, previous history of benign breast diseases, body mass index (BMI), shift work experiences, smoking status, alcohol and tea drinking consumption, and deep fried food and green vegetable consumption.

Results of multivariate logistic regressions for the duration of specific coffee products are presented in Table 5. Consistent with the findings of overall coffee consumption, the duration of consumption of coffee overall was not associated with the risk of breast cancer (AOR = 1.10, 95% CI = 0.80–1.53). However, women who drank instant coffee for more than ten years had an 48% increased risk of breast cancer. This was conflicting to that of in women who drank brewed coffee (AOR = 0.40, 95% CI 0.21–0.77).

**Table 5:** Distribution of duration of specific coffee product and its association with breast cancer risk^a^.

Duration of coffee consumption, n (%)	Controls (*N* = 1 013)	Cases (*N* = 1 156)	Crude OR (95% CI)^bc^	Adjusted OR (95% CI)^bcd^
**Overall coffee**				
Non-habitual coffee drinker	834 (82.3)	917 (79.3)	1.00	1.00
5–10 years	81 (8.0)	107 (9.3)	1.20 (0.89–1.63)	1.15 (0.81–1.63)
>10 years	98 (9.7)	131 (11.3)	1.21 (0.92–1.60)	1.10 (0.80–1.53)
**Instant coffee**				
Non-habitual coffee drinker	900 (89.8)	974 (84.3)	1.00	1.00
5–10 years	52 (5.1)	83 (7.2)	**1.48 (1.03–2.11)**	1.45 (0.97–2.17)
>10 years	61 (6.0)	99 (8.6)	**1.50 (1.08–2.09)**	**1.48 (1.01–2.17)**
Two/Three in one				
Non-habitual coffee drinker	998 (98.5)	1 119 (96.8)	1.00	1.00
5–10 years	6 (0.6)	15 (1.3)	2.27 (0.89–5.88)	2.99 (0.94–9.42)
>10 years	9 (0.9)	22 (1.9)	**2.22 (1.02–4.85)**	2.23 (0.92–5.43)
**Brewed coffee**				
Non-habitual coffee drinker	954 (94.2)	1 112 (96.2)	1.00	1.00
5–10 years	27 (2.7)	20 (1.7)	0.67 (0.38–1.21)	0.66 (0.35–1.26)
>10 years	32 (3.2)	23 (2.0)	0.65 (0.38–1.13)	**0.40 (0.21–0.77)**
Homemade coffee				
Non-habitual coffee drinker	989 (97.6)	1 130 (97.8)	1.00	1.00
5–10 years	13 (1.3)	13 (1.1)	0.87 (0.40–1.90)	0.89 (0.38–2.07)
>10 years	12 (1.2)	13 (1.1)	0.95 (0.43–2.09)	0.62 (0.24–1.61)
Restaurant coffee				
Non-habitual coffee drinker	976 (96.3)	1 138 (98.4)	1.00	1.00
5–10 years	15 (1.5)	7 (0.6)	**0.40 (0.16–0.99)**	0.44 (0.17–1.11)
>10 years	21 (2.1)	10 (0.9)	**0.41 (0.19–0.87)**	**0.26 (0.11–0.64)**
**Others** ^**e**^				
Non-habitual coffee drinker	994 (98.1)	1 130 (97.8)	1.00	1.00
5–10 years	8 (0.8)	11 (1.0)	1.21 (0.49–3.02)	1.04 (0.35–3.10)
>10 years	11 (1.1)	15 (1.3)	1.20 (0.55–2.62)	1.18 (0.51–2.71)

^a^Participants with missing values were excluded from the analysis.

^b^The reference group was defined as the participants who did not have a habitual coffee consumption (i.e., coffee drinking vs. non-coffee drinking; instant coffee drinking vs. non- coffee drinking).

^c^Participants who drank both instant coffee and brewed coffee were excluded in the logistic regression models.

^d^Adjusted for age at interview, educational attainment, family breast cancer history, previous history of benign breast diseases, body mass index (BMI), shift work experiences, smoking status, alcohol and tea drinking consumption, and deep fried food and green vegetable consumption.

^e^Other coffee referred to those who drank decaffeinated coffee or had no preferable coffee products.

Sensitivity analyses were conducted by further including history of hypertension and diabetes mellitus into the multivariate logistic models. As shown in Supplement I–III, the AORs for breast cancer were similar to those obtained from the models without including history of hypertension and diabetes. For instance, the AOR for instant coffee in the model without history of hypertension and diabetes vs. the model with history of hypertension and diabetes was 1.50 (1.10–2.03) vs. 1.52 (95% CI: 1.11–2.07), while the corresponding AOR for brewed coffee was 0.48 (0.28–0.82) vs. 0.52 (95% CI: 0.30–0.90), respectively.

We performed further stratified analyses according to menopausal status (Table 6). Among postmenopausal women, the association for instant coffee, brewed coffee and restaurant coffee retained statistical significance. However, a significantly positive association with the pre-menopausal breast cancer was only observed among those who had habits of drinking two-in-one or three-in-one coffee (AOR = 5.37, 95% CI = 1.14–25.38).

**Table 6:** Association between controls and breast cancers in coffee products consumption stratified by menopausal status^a^.

Characteristics	Pre-menopausal			Post-menopausal		
	Control *n* = 341	All cases *n* = 388	Adjusted OR^bcd^ (95% CI)	Control *n* = 616	All cases *n* = 695	Adjusted OR^bcd^ (95% CI)
Coffee drinking, n (%)	67 (19.6)	78 (20.1)	1.00 (0.65–1.54)	106 (17.2)	149 (21.4)	1.15 (0.83–1.60)
Instant coffee	42 (12.3)	60 (15.5)	1.44 (0.86–2.44)	67 (10.9)	114 (16.4)	**1.52 (1.03–2.25)**
Two/Three in one	3 (0.9)	13 (3.4)	**5.37 (1.14–25.38)**	12 (1.9)	23 (3.3)	1.82 (0.79–4.17)
Brewed coffee	24 (7.0)	18 (4.6)	0.51 (0.21–1.19)	34 (5.5)	23 (3.3)	**0.40 (0.20–0.83)**
Homemade coffee	9 (2.6)	6 (1.5)	0.61 (0.13–2.91)	15 (2.4)	19 (2.7)	0.75 (0.31–1.84)
Restaurant coffee	15 (4.4)	11 (2.8)	0.48 (0.17–1.30)	21 (3.4)	4 (0.6)	**0.13 (0.04–0.49)**
Others^e^	8 (2.3)	8 (2.1)	0.72 (0.25–2.11)	10 (1.6)	18 (2.6)	1.41 (0.57–3.47)

^a^Participants with missing values were excluded from the analysis.

^b^The reference group was defined as the participants who did not have a habitual coffee consumption (i.e., coffee drinking vs. non-coffee drinking; instant coffee drinking vs. non- coffee drinking).

^c^Participants who drank both instant coffee and brewed coffee were excluded in the logistic regression models. ^d^Adjusted for age at interview, educational attainment, family breast cancer history, previous history of benign breast diseases, body mass index (BMI), shift work experiences, smoking status, alcohol and tea drinking consumption, and deep fried food and green vegetable consumption.

^e^Other coffee referred to those who drank decaffeinated coffee or had no preferable coffee products.

## Discussion

This hospital-based case-control study demonstrated the different associations between brewed coffee and instant coffee with breast cancer risk. Women who habitually consumed brewed coffee were associated with 52% lower in the risk of breast cancer, and the inverse association was even predominant among postmenopausal women. In contrast, instant coffee consumption was positively associated with the risk of breast cancer, and a further higher risk was observed among habitual two-in-one or three-in-one coffee drinkers who were pre-menopausal. These findings add additional evidence to the current literature on the association between coffee products and the risk of breast cancer.

We found that the duration and number of cups of brewed coffee consumption were negatively associated with the risk of breast cancer among Hong Kong Chinese women. Women who drank more than one cup of brewed coffee per day and drank for more than ten years were inversely associated with 60% breast cancer risk, respectively. One possible mechanism of this association may be related to the rich polyphenols (such as, caffeic acid and chlorogenic acid) in coffee, which may inhibit the breast cancer related gene *RAR-Beta’s* promoter and methylation^13^. Another mechanism of this negative association between brewed coffee and breast cancer risk may be related to a high level of caffeine intake which was evidently to be associated with a lower level of circulating estrogen^11,14^. A high level of circulating estrogen was found to be associated with a significantly increased risk of breast cancer^15^. Caffeine consumption may reduce total luteal cells and free estradiol level in premenopausal women^14^ but increase the levels of sex hormone-binding globulin in postmenopausal women^11^. In postmenopausal women, the ovaries were not the major source of estrogen, as the estradiol may be produced in a number of extragonadal sites^16^. Sex hormone-binding globulin helps to bind the estradiol and testosterone from number of extragonadal sites which decreases the levels of bioavailable estradiol and testosterone^11^, and the levels of sex hormone-binding globulin were associated with decreased risk of breast cancer among postmenopausal women^17^. Previous epidemiological study also observed an association of caffeine with higher levels of sex hormone-binding globulin and low levels of free testosterone^14^, which explains an inverse association between coffee intake and breast cancer risk through the possible pathway of estrogen among postmenopausal^11^. By contrast, the principal source of estrogen is produced in ovaries among premenopausal women whose estrogen levels are relatively higher than the postmenopausal women^16^, which explains the reduction of estrogen levels induced by the intake of coffee may not affect much on the risk of breast cancer for the premenopausal women. As a result, brewed coffee consumption may decrease the risk of breast cancer via reducing the levels of estrogen among postmenopausal women but not significant in premenopausal women. Consistent with some of the previous studies^4,5,10^, our study also demonstrated a significantly negative association between brewed coffee consumption habits and breast cancer risk in the postmenopausal women. However, one recent meta-analysis study reported a similarly pooled negative association of caffeinated and decaffeinated coffee with breast cancer risk^5^. These negative associations from the meta-analysis may suggest other potential coffee protective substances in coffee or the potential protective effects, which only affects post-menopausal women.

Another putative mechanism for the inverse association of brewed coffee consumption and breast cancer risk was the exposure of diterpene. The levels of diterpene vary among different brewing methods^18^. Higher levels of two specific coffee diterpenes, cafestol and kahweol are more evident in boiled coffee than in filtered coffee^18^. These two diterpenes produced the biochemical effects such as induction of conjugating enzymes and increased protein expression in cellular antioxidant defense, which may result in a reduction of the genotoxicity of several carcinogens, such as 7,12-Dimethylbenz(a)anthracene, benzopyrene and 2-amino-1-methyl-6-phenylimidazo(4,5-b)pyridine^19,20^. The anti-carcinogenic activity of cafestrol and kahweol reduces the expression and inhibits enzymatic activity of phase I enzyme for carcinogen activation, and induces phase II enzymes in carcinogen detoxification^19^. Kahweol increased the production of reactive oxygen species and their cytotoxicity in breast cancer cells, which enhances apoptosis of breast tumor cells^20^. One prospective cohort study conducted in Vasterbotten reports a significantly decreased risk of breast cancer among women who habitually drink boiled coffee, but a weaker positive association was suggested in women with habitual filtered coffee drinking^7^. Among our participants who have habitual restaurant coffee drinking habits, approximately 90% of them drank their coffee in Hong Kong-style diners (cha chaan teng) or Hong Kong-style fast food restaurants. Such restaurants serve boiled coffee, which may be a plausible explanation of the stronger association with breast cancer observed in restaurant coffee than that of the homemade coffee.

Intriguingly, there is a positive association of instant coffee consumption with the risk of breast cancer, and a clear exposure– response relationship of cups of instant coffee was revealed with breast cancer risk. These instant coffees contain less coffee but more additives, such as non-diary creamer, sugar and stabilizers to maintain the coffee texture, taste and smell. However, these additives may also contain hydrogenated fats and/or trans-saturated fats that may increase breast cancer risk^21^. In addition, habitual instant coffee drinkers tend to have lower serum levels of high density lipoprotein (HDL) cholesterol^22^. One population-based study in Troms reported that low serum HDL cholesterol was associated with increased levels of estradiol concentration^23^, which may further increase the risk of breast cancer among the instant coffee drinkers.

To the best of our knowledge, this is the first study reporting the association between different coffee products (i.e. instant coffee and brewed coffee) and breast cancer risk in Hong Kong Chinese women. Information on coffee consumption habits were amassed, such as the type of coffee products, the number of cups and years consumed. Results of our study propose that instant coffee may be a novel breast cancer risk factor. We recruited our participants from three large public hospitals, covering around 8.5% of those who had breast cancer in Hong Kong’s population, with a high response rate (88.4%). We further re-interviewed 158 cases and 153 controls at least one month after the initial recruitment and showed good overall test-retest reliability for tea drinking (Kappa consistency rate of 83%). However, the major limitations of our study do exist. We did not collect the information regarding the preparation method of coffee (e.g., filtered, boiled), in which the content of compounds and chemicals including caffeine may vary. Misclassification of the concentration of caffeine and other components in the different coffee product may also be a concern, as we do not have direct measurements of each content. Furthermore, the issue of chance cannot be entirely excluded in subgroup analysis for the associations between different coffee products and breast cancer, since the 95% CI was relatively broad. Despite the mechanism for instant coffee on the risk of breast cancer is not yet clear and the significant results of these associations could be due by chance, the consistently positive dose-response relationships between daily amount of consumption or duration of instant coffee drinking with breast cancer risk increase the plausibility of our findings. Generally, the concern of the recall and/or interviewer bias cannot be eliminated in case-control studies, and thus we used a standardized approach to interview all participants and interviewed the controls within 6 month after the cases were recruited, which in turn reduced the potential recall and interview bias. In addition, we compared the findings between cases recruited from Department of Survey (cases were interviewed before biopsy) with those from Department of Clinical Oncology (the diagnosis of breast cancer had been known), and they were similar which further supports that the potential recall and/or interveiw bias of our study should not be a main concern. Selection bias of breast cases may be a concern as all cases of our study came from three hospitals. We found a similar proportion of incident breast cancer in different age groups to those obtained from the Hong Kong Cancer Registry from 2011 to 2016 (i.e. the last updated year)^24^, indicating a good comparability between our cases and the general population. Controls recruited from the hospitals may not well represent the general population; however, we recruited controls with a variety of disease types from the same hospitals that were not related to any type of breast diseases. Previous hospital-based case-control studies provide evidence that a similar magnitude of risk was obtained between using the general population controls and hospital controls with a variety of disease types^25^. Thus, the potential selection bias, if presents in this study, should not be a major issue.

In summary, this study revealed that breast cancer among Chinese women in Hong Kong was inversely associated with the consumption of brewed coffee but positively related to the intake of instant coffee. Nevertheless, it should be noted that breast cancer cases in this study showed a low prevalence of habitual coffee consumption. Future larger analytic studies with different population subsets and types of coffee products are warranted.

## Methods

### Study population and design

Details of the study design and subject characteristics have been described elsewhere^26^. We conducted a hospital-based case-control study in three Hong Kong public hospitals from November 2011 to January 2018. Trained interviewers conducted a face-to-face interviews with a standardized questionnaire and obtained a written informed consent form. Eligible cases were Chinese women aged 24–84 years-old who were newly diagnosed as having primary breast cancer (International Classification of Disease, Tenth Revision, code 50) within three months prior to the interview. We matched each case with one control by 5-year age group who did not have any conditions pertaining to breast cancer, and interviewed them within 6 month after the cases were recruited. We recruited our controls with a broad spectrum of diagnoses from the Department of Medicine or Surgery from the same hospital of the cases came from. We excluded cases and controls with prior history of physician-diagnosed cancer in any site. This study was approved by the Joint Chinese University of Hong Kong-New Territories East Cluster Clinical Research Ethics Committees and the Kowloon West Cluster, and was strictly compliance with local law and the Declaration of Helsinki.

A standardized questionnaire gathered information on socio-demographic characteristics, tobacco smoking, alcohol drinking, dietary habits such as tea and coffee consumption patterns, hormone-related factors including menopausal status, anthropometric risk factors, history of chronic diseases including breast diseases, family cancer history, physical activity, and occupational history including shift work.

We asked each participant to report their coffee consumption patterns. Participants who drank any type of coffee product at least once a week over 5 years prior to our recruitment time frame were identified as habitual coffee drinkers, whereas those who did not have such a habit were defined as non-habitual coffee drinkers. We further requested the habitual coffee drinkers to provide information on the coffee product that they consumed (instant coffee, brewed coffee, etc.), number of cups (defined as 250 ml per time) and years of coffee consumption. The category of ‘instant coffee’ refers to the coffee that is soluble and ready to drink. For instance, three-in-one instant coffee is the mixture of soluble coffee, non-dairy creamer and sugar. Brewed coffee refers to the coffee that requires different brewing processes before consumption, such as being boiled and filtered. For the participants who have switched their habitual consumption type of coffee (i.e. from an instant coffee drinker to a brewed coffee drinker, or vice versa), the recent type of coffee comsumption were defined as their habitual consumption types; if the participants’ habits of recent type of coffee comsuption were less than 5 years, they were then defined as the previous type coffee drinkers.

### Statistical analysis

Independent t-tests and chi-square test were performed to compare the differences between cases and controls for the continuous and categorical variables, respectively. We estimated the adjusted odds ratios (AOR) and 95% confidence interval (95% CI) for the associations between different coffee products and breast cancer with an unconditional multivariate logistic regression model. The reference group was defined as the participants who did not have a habitual coffee consumption (i.e., coffee drinking vs. non-coffee drinking; instant coffee drinking vs. non- coffee drinking). We adjusted for potential confounders including the participant’s age, educational attainment, family breast cancer history, previous history of benign breast diseases, menopausal status, body mass index (BMI), shift work, smoking habits, alcohol and tea drinking consumption, and deep fried food and green vegetable consumption. Further stratified analyses were conducted according to their menopausal status. Postmenopausal status was determined if the participants reported their menstrual periods stopped more than a year; otherwise, pre-menupausal status was defined. All statistical analyses were conducted with SPSS 20.0 for Windows (SPSS, Chicago, IL, USA), and two-sided p-value less than 0.05 was considered statistically significant.

## Supplementary information

Distribution of specific coffee product among habitual coffee consumption and its association with breast cancer risk

## Data Availability

The results and materials described in the article, and the relevant raw data related to coffee drinking can be freely available upon the request to the corresponding author.

## References

[1] WHO. *Breast cancer*, http://www.who.int/cancer/prevention/diagnosis-screening/breast-cancer/en/ (2018).

[2] Statista. *Coffee*, https://www.statista.com/outlook/30010000/118/coffee/hong-kong (2018).

[3] Loomis D (2016). Carcinogenicity of drinking coffee, mate, and very hot beverages. Lancet Oncol.

[4] Ganmaa D (2008). Coffee, tea, caffeine and risk of breast cancer: a 22-year follow-up. Int J Cancer.

[5] Jiang W, Wu Y, Jiang X (2013). Coffee and caffeine intake and breast cancer risk: an updated dose-response meta-analysis of 37 published studies. Gynecol Oncol.

[6] Lowcock EC, Cotterchio M, Anderson LN, Boucher BA, El-Sohemy A (2013). High coffee intake, but not caffeine, is associated with reduced estrogen receptor negative and postmenopausal breast cancer risk with no effect modification by CYP1A2 genotype. Nutr Cancer.

[7] Nilsson LM, Johansson I, Lenner P, Lindahl B, Van Guelpen B (2010). Consumption of filtered and boiled coffee and the risk of incident cancer: a prospective cohort study. Cancer Causes Control.

[8] Fagherazzi G, Touillaud MS, Boutron-Ruault MC, Clavel-Chapelon F, Romieu I (2011). No association between coffee, tea or caffeine consumption and breast cancer risk in a prospective cohort study. Public Health Nutr.

[9] Hashibe M (2015). Coffee, tea, caffeine intake, and the risk of cancer in the PLCO cohort. Br J Cancer.

[10] Lafranconi Alessandra, Micek Agnieszka, De Paoli Paolo, Bimonte Sabrina, Rossi Paola, Quagliariello Vincenzo, Berretta Massimiliano (2018). Coffee Intake Decreases Risk of Postmenopausal Breast Cancer: A Dose-Response Meta-Analysis on Prospective Cohort Studies. Nutrients.

[11] Ferrini RL, Barrett-Connor E (1996). Caffeine intake and endogenous sex steroid levels in postmenopausal women. The Rancho Bernardo Study. Am J Epidemiol.

[12] Fish, I. *30% of instant coffee samples failed biotoxicity test and some contain 90% additives Vitargent publishes safe to buy list of instant coffee rated “Green Fish”*, https://www.fishqc.com/en/information-72 (2017).

[13] Lee WJ, Zhu BT (2006). Inhibition of DNA methylation by caffeic acid and chlorogenic acid, two common catechol-containing coffee polyphenols. Carcinogenesis.

[14] Kotsopoulos J, Eliassen AH, Missmer SA, Hankinson SE, Tworoger SS (2009). Relationship between caffeine intake and plasma sex hormone concentrations in premenopausal and postmenopausal women. Cancer.

[15] Fuhrman BJ (2012). Estrogen metabolism and risk of breast cancer in postmenopausal women. J Natl Cancer Inst.

[16] Simpson ER (2003). Sources of estrogen and their importance. J Steroid Biochem Mol Biol.

[17] Key T (2002). Endogenous sex hormones and breast cancer in postmenopausal women: reanalysis of nine prospective studies. J Natl Cancer Inst.

[18] Gross G, Jaccaud E, Huggett AC (1997). Analysis of the content of the diterpenes cafestol and kahweol in coffee brews. Food Chem Toxicol.

[19] Cavin C (2002). Cafestol and kahweol, two coffee specific diterpenes with anticarcinogenic activity. Food Chem Toxicol.

[20] Cardenas C, Quesada AR, Medina MA (2014). Insights on the antitumor effects of kahweol on human breast cancer: decreased survival and increased production of reactive oxygen species and cytotoxicity. Biochem Biophys Res Commun.

[21] Wang J, John EM, Horn-Ross PL, Ingles SA (2008). Dietary fat, cooking fat, and breast cancer risk in a multiethnic population. Nutr Cancer.

[22] Kim HJ, Cho S, Jacobs DR, Park K (2014). Instant coffee consumption may be associated with higher risk of metabolic syndrome in Korean adults. Diabetes Res Clin Pract.

[23] Furberg AS (2005). Metabolic and hormonal profiles: HDL cholesterol as a plausible biomarker of breast cancer risk. The Norwegian EBBA Study. Cancer epidemiology, biomarkers & prevention: a publication of the American Association for Cancer Research, cosponsored by the American Society of Preventive Oncology.

[24] Hong Kong Cancer Registry Hospital Authority. *Cancer Statistics Query Systems*, http://www3.ha.org.hk/cancereg/allages.asp (2018).

[25] Li L, Zhang M, Holman D (2011). Population versus hospital controls for case-control studies on cancers in Chinese hospitals. BMC medical research methodology.

[26] Li M (2017). Nighttime eating and breast cancer among Chinese women in Hong Kong. Breast Cancer Res.

