# Supplementary material for: Associations between Coffee Products and Breast Cancer Risk: a Case-Control study in Hong Kong Chinese Women
Source: Sci Rep. 2019 Sep 3;9:12684. doi: 10.1038/s41598-019-49205-x (PMC6722060; doi:10.1038/s41598-019-49205-x)
Supplement: Supplementary file 1 — Distribution of specific coffee product among habitual coffee consumption and its association with breast cancer risk [file 41598_2019_49205_MOESM1_ESM.docx]

**Associations between Coffee Products and Breast Cancer Risk: a Case-Control study in Hong Kong Chinese Women**

Priscilla Ming Yi LEE^1†^, Wing Cheong CHAN^2†^, Carol Chi-hei KWOK^3^, Cherry WU^4^, Sze-hong LAW^5^, Koon-ho TSANG^6^, Wai-cho YU^7^, Yiu-cheong YEUNG^7^, Lydia Dai Jia CHANG^8^, Carmen Ka Men WONG^1^, Feng WANG^1^, Lap Ah TSE^1*^

**Affiliations** ^1^ The Jockey Club School of Public Health and Primary Care, Faculty of Medicine, The Chinese University of Hong Kong, Hong Kong, People's Republic of China
^2^ Department of Surgery, North District Hospital, Hong Kong, People's Republic of China
^3^ Department of Oncology, Princess Margaret Hospital, Hong Kong, People's Republic of China  ^4^ Department of Pathology, North District Hospital, Hong Kong, People's Republic of China
^5^ Department of Surgery, Yan Chai Hospital, Hong Kong, People's Republic of China
^6^ Department of Pathology, Yan Chai Hospital, Hong Kong, People's Republic of China
^7^ Department of Medicine, Princess Margaret Hospital, Hong Kong, People's Republic of China
^8^ Department of Biological Sciences, University of Toronto Scarborough, Toronto, Canada

**Supplement Table Legends**

Supplement I. Distribution of specific coffee product among habitual coffee consumption and its association with breast cancer risk

Supplement II. Distribution of number of cups of coffee products and its association with breast cancer riska

Supplement III. Distribution of duration of specific coffee product and its association with breast cancer risk

Supplement I. Distribution of specific coffee product among habitual coffee consumption and its association with breast cancer risk^a^

| Variables | Controls  (*N*=1 013) | Cases  (*N*=1 156) | Crude OR  (95%CI) ^bc^ | Adjusted OR (95%CI) ^bcd^ |
| --- | --- | --- | --- | --- |
| Coffee drinking, n (%) | 179 (17.7) | 238 (20.6) | 1.21 (0.97-1.50) | 1.16 (0.89-1.52) |
| Instant coffee | 113 (11.2) | 182 (15.7) | **1.52 (1.18-1.99)** | **1.52 (1.11-2.09)** |
| Two/Three in one | 15 (1.5) | 37 (3.2) | **2.24 (1.22-4.11)** | **2.65 (1.26-5.59)** |
| Brewed coffee | 59 (5.8) | 43 (3.7) | **0.57 (0.36-0.90)** | **0.52 (0.30-0.90)** |
| Homemade coffee | 25 (2.5) | 26 (2.2) | 0.86 (0.45-1.65) | 0.83 (0.38-1.83) |
| Restaurant coffee | 36 (3.6) | 17 (1.5) | **0.39 (0.20-0.76)** | **0.31 (0.14-0.68)** |
| Others ^d^ | 19 (1.9) | 26 (2.2) | 1.24 (0.68-2.26) | 1.00 (0.49-2.02) |

^a^ Participants with missing values were excluded from the analysis.

^b^ The reference group was defined as the participants who did not have a habitual coffee consumption on specific (i.e., coffee drinking vs. non-coffee drinking; instant coffee drinking vs. non-coffee drinking).
^c^ Participants who drank both instant coffee and brewed coffee were excluded in the logistic models.

^d^ Adjusted for age at interview, educational attainment, family breast cancer history, previous history of benign breast diseases, body mass index (BMI), shift work experiences, smoking status, alcohol and tea drinking consumption, and deep fried food and green vegetable consumption, history of hypertension and diabetes mellitus.
^e^ Other coffee referred to those who drank decaffeinated coffee or had no preferable coffee products

Supplement II. Distribution of number of cups of coffee products and its association with breast cancer risk^a^

| Coffee drinking, n (%) | Controls  (*N*=1 013) | Cases  (*N*=1 156) | Crude OR  (95%CI) ^bc^ | Adjusted OR (95%CI) ^bcd^ |
| --- | --- | --- | --- | --- |
| Overall coffee |  |  |  |  |
| Non-habitual coffee drinker | 834 (82.3) | 918 (79.4) | 1.00 | 1.00 |
| <1 cup/day | 51 (5.0) | 77 (6.7) | 1.37 (0.95-1.98) | 1.24 (0.80-1.92) |
| ≥1 cup/day | 128 (12.6) | 161 (13.9) | 1.14 (0.89-1.47) | 1.12 (0.83-1.52) |
| Instant coffee |  |  |  |  |
| Non-habitual coffee drinker | 900 (88.8) | 974 (84.3) | 1.00 | 1.00 |
| <1 cup/day | 37 (3.7) | 63 (5.4) | **1.55 (1.02-2.35)** | 1.48 (0.91-2.43) |
| ≥1 cup/day | 76 (7.5) | 119 (10.3) | **1.42 (1.05-1.93)** | **1.44 (1.00-2.07)** |
| Two/Three in one |  |  |  |  |
| Non-habitual coffee drinker | 998 (98.5) | 1 119 (96.8) | 1.00 | 1.00 |
| <1 cup/day | 4 (0.4) | 10 (0.9) | 2.27 (0.71-7.27) | 3.06 (0.64-14.64) |
| ≥1 cup/day | 11(1.1) | 27 (2.3) | **2.23 (1.10-4.52)** | **2.66 (1.14-6.22)** |
| Brewed coffee |  |  |  |  |
| Non-habitual coffee drinker | 954 (94.2) | 1 112 (96.3) | 1.00 | 1.00 |
| <1 cup/day | 24 (2.4) | 25 (2.2) | 0.95 (0.54-1.67) | 0.79 (0.40-1.54) |
| ≥1 cup/day | 35 (3.5) | 18 (1.6) | **0.47 (0.26-0.83)** | **0.45 (0.23-0.89)** |
| Homemade coffee |  |  |  |  |
| Non-habitual coffee drinker | 889 (97.7) | 902 (97.5) | 1.00 | 1.00 |
| <1 cup/day | 10 (1.0) | 13 (1.1) | 1.18 (0.52-2.71) | 1.32 (0.50-3.50) |
| ≥1 cup/day | 15 (1.5) | 13 (1.1) | 0.79 (0.37-1.66) | 0.73 (0.29-1.82) |
| Restaurant coffee |  |  |  |  |
| Non-habitual coffee drinker | 977 (96.4) | 1 139 (98.5) | 1.00 | 1.00 |
| <1 cup/day | 17 (1.7) | 12 (1.0) | 0.64 (0.30-1.35) | 0.43 (0.18-1.05) |
| ≥1 cup/day | 19 (1.9) | 5 (0.4) | **0.24 (0.09-0.64)** | **0.28 (0.10-0.79)** |
| Others ^e^ |  |  |  |  |
| Non-habitual coffee drinker | 897 (98.2) | 898 (97.4) | 1.00 | 1.00 |
| <1 cup/day | 6 (0.6) | 12 (1.0) | 1.82 (0.68-4.86) | 1.65 (0.45-6.03) |
| ≥1 cup/day | 13 (1.3) | 14 (1.2) | 0.98 (0.46-2.09) | 0.79 (0.34-1.81) |

^a^ Participants with missing values were excluded from the analysis.

^b^ The reference group was defined as the participants who did not have a habitual coffee consumption (i.e., coffee drinking vs. non-coffee drinking; instant coffee drinking vs. non-instant coffee drinking).

^c^ Participants who drank both instant coffee and brewed coffee were excluded in the logistic models.

^d^ Adjusted for age at interview, educational attainment, family breast cancer history, previous history of benign breast diseases, body mass index (BMI), shift work experiences, smoking status, alcohol and tea drinking consumption, and deep fried food and green vegetable consumption, history of hypertension and diabetes mellitus.
^e^ Other coffee referred to those who drank decaffeinated coffee or had no preferable coffee products.

Supplement III. Distribution of duration of specific coffee product and its association with breast cancer risk^a^

| Duration of coffee drinking, n (%) | Controls  (*N*=1 013) | Cases  (*N*=1 156) | Crude OR  (95%CI) ^bc^ | Adjusted OR (95%CI) ^bcd^ |
| --- | --- | --- | --- | --- |
| Overall coffee |  |  |  |  |
| Non-habitual coffee drinker | 834 (82.3) | 917 (79.3) | 1.00 | 1.00 |
| 5-10 years | 81 (8.0) | 107 (9.3) | 1.20 (0.89-1.63) | 1.22 (0.85-1.76) |
| >10 years | 98 (9.7) | 131 (11.3) | 1.21 (0.92-1.60) | 1.09 (0.78-1.53) |
| Instant coffee |  |  |  |  |
| Non-habitual coffee drinker | 900 (89.8) | 974 (84.3) | 1.00 | 1.00 |
| 5-10 years | 52 (5.1) | 83 (7.2) | **1.48 (1.03-2.11)** | 1.48 (0.97-2.26) |
| >10 years | 61 (6.0) | 99 (8.6) | **1.50 (1.08-2.09)** | 1.43 (0.96-2.13) |
| Two/Three in one |  |  |  |  |
| Non-habitual coffee drinker | 998 (98.5) | 1 119 (96.8) | 1.00 | 1.00 |
| 5-10 years | 6 (0.6) | 15 (1.3) | 2.27 (0.89-5.88) | 2.97 (0.92-9.55) |
| >10 years | 9 (0.9) | 22 (1.9) | **2.22 (1.02-4.85)** | 2.44 (0.94-6.36) |
| Brewed coffee |  |  |  |  |
| Non-habitual coffee drinker | 954 (94.2) | 1 112 (96.2) | 1.00 | 1.00 |
| 5-10 years | 27 (2.7) | 20 (1.7) | 0.67 (0.38-1.21) | 0.77 (0.40-1.50) |
| >10 years | 32 (3.2) | 23 (2.0) | 0.65 (0.38-1.13) | **0.46 (0.23-0.90)** |
| Homemade coffee |  |  |  |  |
| Non-habitual coffee drinker | 989 (97.6) | 1 130 (97.8) | 1.00 | 1.00 |
| 5-10 years | 13 (1.3) | 13 (1.1) | 0.87 (0.40-1.90) | 1.24 (0.50-3.04) |
| >10 years | 12 (1.2) | 13 (1.1) | 0.95 (0.43-2.09) | 0.71 (0.26-1.91) |
| Restaurant coffee |  |  |  |  |
| Non-habitual coffee drinker | 976 (96.3) | 1 138 (98.4) | 1.00 | 1.00 |
| 5-10 years | 15 (1.5) | 7 (0.6) | **0.40 (0.16-0.99)** | 0.42 (0.16-1.14) |
| >10 years | 21 (2.1) | 10 (0.9) | **0.41 (0.19-0.87)** | **0.31 (0.13-0.77)** |
| Others ^e^ |  |  |  |  |
| Non-habitual coffee drinker | 994 (98.1) | 1 130 (97.8) | 1.00 | 1.00 |
| 5-10 years | 8 (0.8) | 11 (1.0) | 1.21 (0.49-3.02) | 1.01 (0.33-3.13) |
| >10 years | 11 (1.1) | 15 (1.3) | 1.20 (0.55-2.62) | 0.96 (0.40-2.33) |

^a^ Participants with missing values were excluded from the analysis.

^b^ The reference group was defined as the participants who did not have a habitual coffee consumption (i.e., coffee drinking vs. non-coffee drinking; instant coffee drinking vs. non-instant coffee drinking).

^c^ Participants who drank both instant coffee and brewed coffee were excluded in the logistic models.

^d^ Adjusted for age at interview, educational attainment, family breast cancer history, previous history of benign breast diseases, body mass index (BMI), shift work experiences, smoking status, alcohol and tea drinking consumption, and deep fried food and green vegetable consumption, history of hypertension and diabetes mellitus.

^e^ Other coffee referred to those who drank decaffeinated coffee or had no preferable coffee products.
